# Normal Alpha-Fetoprotein Hepatocellular Carcinoma: Are They Really Normal?

**DOI:** 10.3390/jcm8101736

**Published:** 2019-10-19

**Authors:** Chao-Wei Lee, Hsin-I Tsai, Wei-Chen Lee, Shu-Wei Huang, Cheng-Yu Lin, Yi-Chung Hsieh, Tony Kuo, Chun-Wei Chen, Ming-Chin Yu

**Affiliations:** 1 Division of General Surgery, Department of Surgery, Linkou Chang Gung Memorial Hospital, Taoyuan City 333, Taiwan; alanchaoweilee@hotmail.com (C.-W.L.); weichen@adm.cgmh.org.tw (W.-C.L.); 2 Graduate Institute of Clinical Medical Sciences, Chang Gung University, Taoyuan City 333, Taiwan; tsaic@hotmail.com; 3 College of Medicine, Chang Gung University, Taoyuan City 333, Taiwan; 8705002@cgmh.org.tw (S.-W.H.); 8805035@cgmh.org.tw (C.-Y.L.); cutebuw@yahoo.com.tw (Y.-C.H.); B9302028@cgmh.org.tw (T.K.); 4 Department of Anesthesiology, Linkou Chang Gung Memorial Hospital, Taoyuan City 333, Taiwan; 5 Department of Gastroenterology and Hepatology, Linkou Chang Gung Memorial Hospital, Taoyuan City 333, Taiwan

**Keywords:** hepatocellular carcinoma, hepatoma, normal alpha-fetoprotein, glypican 3

## Abstract

Introduction: serum alpha-fetoprotein (AFP) was routinely employed as a tumor marker for screening, diagnosis, and treatment follow-up of hepatocellular carcinoma (HCC). However, a substantial proportion of HCC patients had normal AFP level even at an advanced disease status. Few studies to date had tried to explore the nature and behavior of this normal AFP HCC (N-HCC). The purpose of this study was to investigate the clinicopathological characteristics and survival outcome of N-HCC after operation. In addition, potential tumor markers for N-HCC were also sought in an attempt to augment diagnostic ability. Methods: between 2005 and 2015, patients with hepatocellular carcinoma who were treated with hepatectomy in Chang Gung Memorial Hospital Linkou branch were divided into two groups according to their preoperative serum AFP level (<15 ng/mL: NHCC; ≥15 ng/mL: abnormal AFP HCC (A-HCC)). Patient demographic data and clinicopathological variables were collected. Kaplan–Meier and Cox regression multivariate analyses were performed to identify significant risk factors for disease-free survival (DFS) and overall survival (OS) for N-HCC. ELISA and immunohistochemical (IHC) studies were employed to determine the diagnostic accuracy of various tumor markers. Results: a total of 1616 patients (78% male) who underwent liver resection for HCC were included in this study. Of them, 761 patients (47.1%) were N-HCC. N-HCC patients were significantly older with more comorbidities and less hepatitis virus infections. Furthermore, N-HCC had fewer early recurrences (49.6% vs. 60.8%, *p* < 0.001) and better DFS (44.6 months vs. 23.6 months, *p <* 0.001) and OS (94.5 months vs. 81.7 months, *p* < 0.001). Both ELISA and IHC studies demonstrated that glypican-3 (GPC3) would be a promising diagnostic tumor marker for N-HCC. Conclusion: N-HCC patients were significantly older and had less hepatitis virus infections or cirrhosis. Their tumors tended to be smaller, less vascular invaded, and well-differentiated. The carcinogenesis of N-HCC may thus not be identical to that of typical HCC. GPC3 would be a promising tumor marker for diagnosing N-HCC. Further study is warranted to validate our findings.

## 1. Introduction

Hepatocellular carcinoma (HCC) is the most common primary malignancy of the liver with an estimated annual death incidence of approximately 700,000 worldwide [1]. In Taiwan, it is the second most common cause of cancer death and causes more than 8000 deaths each year [2]. Viral hepatitis, chronic liver disease, and liver cirrhosis are most common etiologies of HCC. Although curative treatments for early-stage lesions have been improved dramatically, few alternatives exist for late-stage HCC [3]. The vast majority of HCC, unfortunately, is diagnosed at later stage, resulting in its dismal prognosis [4,5]. Effective screening and accurate early diagnosis, subsequently, are mandatory to optimize the outcome of patients with HCC. The diagnosis of HCC nowadays relies primarily on serum biomarkers and radiologic examinations. Common radiologic examinations employed include ultrasonography (US), computed tomography [6], angiography, and magnetic resonance imaging (MRI). However, the nature of operator-dependence for US and contrast/radiation exposure for CT/Angiography/MRI has limited the value of these tests for regular screening and early diagnosis. Therefore, in order to achieve early diagnosis and improve clinical outcomes, the identification of a reliable serum biomarker or a combination of markers is of paramount importance.

Alpha-fetoprotein (AFP), an oncofetal glycoprotein normally expressed in fetus, is currently the most widely used tumor marker for HCC. Its cellular function in adult humans still remains to be determined. In healthy adults, serum AFP level typically falls into the range of 5–10 ng/mL [7]. On the other hand, an elevated serum level of AFP is frequently associated with HCC or other liver diseases. Studies have shown that an AFP level above 400 ng/mL can generally be considered as diagnostic for HCC [3]. AFP, as a result, is frequently adopted as a diagnostic tool for HCC in high risk patients. However, AFP levels below 100 ng/mL are less specific since slightly elevated AFP can also be observed in patients with chronic hepatitis [4]. Moreover, studies have shown that about 40% of HCC had normal AFP levels [4,8,9]. This striking figure alerts clinicians the necessity to explore a more sensitive and specific biomarker for the early diagnosis of this subset of HCC. In addition, few studies to date had tried to explore the nature and behavior of this normal AFP HCC (N-HCC). Although previous studies have already identified elevated AFP to be a robust predictor of poor survival for HCC [10], and CLIP staging system also allocated AFP > 400 ng/mL to be an independent prognostic indicator [11], no studies so far have analyzed the clinical features or survival outcome of N-HCC after hepatectomy. Whether this “normal” AFP HCC is merely an HCC with “lower” or “normal” AFP production or it actually represents a distinct or “abnormal” subtype of HCC is still undetermined. 

Glypican-3 (GPC3) is a member of the heparin sulfate proteoglycans family and bound to the external surface of the plasma membrane by a glycosylphosphatidylinositol bond (GPI) [12]. It is a 70-kDa core protein encoded by the GPC3 gene located on the human X chromosome (Xq26). GPC3, like AFP, is an oncofetal protein expressed only in the placenta and fetal tissues [13]. It regulates cell proliferation and growth by interaction with Wnt signaling and insulin-like growth factor-2 [14,15]. Recently, GPC3 was found to be overexpressed in more than 80% of HCC and was proposed to be a promising tumor marker for the diagnosis of HCC [4,13,16,17]. In addition to GPC3, secreted phophoprotein 1 (SPP1), vascular endothelial growth factor (VEGF), insulin-like growth factor-1 (IGF-1), and hepatocyte growth factor (HGF) have all been implicated as potential serum markers for the diagnosis of HCC [18,19,20,21,22,23]. Nevertheless, despite these remarkable findings, few studies had tried to investigate the diagnostic performance of these markers for HCC with low or normal AFP [24]. Therefore, in addition to investigate the clinicopathological characteristics and survival outcome of N-HCC after the operation, the current study would also compare the diagnostic accuracy of various serum markers for N-HCC.

## 2. Materials and methods

### 2.1. Patients

Under the approval of Institutional Review Boards (CGMH IRB No: 100-4268B and 201600359B0) of Chang Gung Memorial Hospital (CGMH), we retrospectively reviewed patients with HCC who were treated with curative hepatectomy by our surgical team at Linkou CGMH between 2005 and 2015. Exclusion criteria were patients who had distant metastases before operation, who underwent only exploratory laparotomy for liver tumor biopsy, who did not have detailed preoperative/intraoperative clinical records, or who did not have regular postoperative out-patient follow-up. A total of 1616 patients were enrolled and divided into two groups according to their preoperative serum AFP level. The AFP levels were determined by the central laboratory of Linkou CGMH. Our lab employed ARCHITECT AFP Reagent Kit (7K67) (Laboratories, Abbott Park, IL 60064 USA) for the measurement of AFP from 2005. In their large scale study, about 99.5% of healthy subjects had their AFP levels less than 13.4 ng/mL; our hospital set the AFP cutoff at 15 ng/mL as a result. The current study adopted this value and categorized HCC into two groups. Patients with normal preoperative AFP level (<15 ng/mL) were classified as normal AFP HCC group (N-HCC) while those with elevated preoperative AFP level (≥15 ng/mL) were considered abnormal AFP HCC group (A-HCC). All of the demographics, surgical, and perioperative data were reviewed and compared. The study end date was 31 December 2015. Tumor staging was based on the American Joint Committee on Cancer (AJCC) TNM staging system for HCC.

### 2.2. Preoperative Assessment

The diagnosis of HCC was established by characteristic features on imaging by either triphasic computed tomography (CT), magnetic resonance imaging (MRI), hepatic arteriography, and/or a serum α-fetoprotein (AFP) level greater than 200 ng/mL. Resection criteria included absence of distant metastasis, no main trunk portal vein thrombosis, technically operable tumor site and adequate future liver remnant. Child-Pugh classification was routinely evaluated preoperatively. Indocyanine green retention test (ICG-15) was assessed in cirrhotic patients or those who were going to receive major operation. A previous study identified an indocyanine green retention at 15 min (ICG-15) of less than 14% as the safety limit for major hepatic resection [25] In our institute, an ICG-15 ≤ 10% was the prerequisite for major hepatic resection. On the other hand, in patients with higher ICG-15, extensive hepatectomy could also be performed if the liver functional reserve was satisfactory and the size of the future liver remnant was considered adequate according to preoperative CT and intraoperative assessment [26].

### 2.3. Blood Sampling and Assays

To search for potential serum tumor markers for the detection and diagnosis of N-HCC, 147 HCC patients within this entire cohort were enrolled. Among them, 74 patients (50.3%) had normal AFP levels. Another 10 healthy subjects were recruited as normal control. Under informed consent, their blood samples were drawn preoperatively and centrifuged immediately at 1500× *g* for 10 min. The sera were aliquoted and stored at −80 °C for batch analysis. Serum biomarkers were measured using an enzyme-linked immunosorbent assay (ELISA) kit (DuoSet ELISA, R&D Systems; Minneapolis, MN, USA). 

### 2.4. Immunohistochemistry

To study the expressions of various markers, formalin-fixed and paraffin-embedded resection specimens of those patients who had their serum examined by ELISA were retrieved, sectioned to 4 μm in thickness and de-paraffininzed, rehydrated, and processed for antigen retrieval. We included 114 patients. The slides were further incubated with appropriate dilutions of the selected antibodies at room temperature for 1 h. After incubation, the slides were washed three times in phosphate-buffered saline (PBS), incubated with a horse reddish peroxidase conjugated antibody polymer (Zymed) at room temperature for 10 min, and were then developed by treatment with 3,3′-diaminobenzidine (Roche) at room temperature for 10 min.

### 2.5. Definition and Statistical Analysis

Preoperative symptoms included patients presenting with jaundice, anemia, ascites, or palpable mass when establishing the diagnosis. Major operation defined hepatectomy involved three or more liver segments [27]. Major surgical complications comprised grade III and grade IV surgical complications [28]. For statistical analysis, Fisher’s exact test and Pearson’s χ^2^ test were used to analyze categorical data. Student’s *t* test and Mann–Whitney U test were used to analyze continuous variables. Significant variables in univariate analysis were then subjected into a stepwise cox regression multivariate analyses. The Kaplan–Meier method was employed for survival analysis and the results were compared with the log-rank test. The receiver operating characteristic (ROC) curve was developed to determine the sensitivity and specificity of individual serum maker. The area under the curve (AUC) value was compared between these markers. All calculations were performed with SPSS for windows (SPSS Inc., Chicago, IL, USA). Two-tailed *P*-values less than 0.05 were considered statistically significant.

## 3. Results

### 3.1. Clinical-Pathological Characteristics of N-HCC Versus A-HCC

A total of 1616 patients with HCC underwent curative hepatectomy during the study period. The median follow-up time was 39.5 months. Among them, 761 (47.1%) patients had AFP levels less than 15 ng/mL (N-HCC), 312 (19.3%) had AFP levels between 15 and 100 ng/mL, 184 (11.4%) had AFP between 100 and 400 ng/mL, and the remaining 359 (22.2%) patients had AFP greater than 400 ng/mL. An elevated AFP (A-HCC) was demonstrated in 855 patients (52.9%) in the current study. After statistical analysis, we found that N-HCC patients were generally older (*P* = 0.020) with male predominance (*P* = 0.001), having more co-morbidities such as diabetes mellitus (*P* < 0.001), and having less hepatitis B virus (HBV) infection (*P* < 0.001) (Table 1). Interestingly, almost 20% of N-HCC patients had neither HBV nor HCV infections, as compared to only 9% in the A-HCC group (*P* < 0.001). On the other hand, the ICG-15 level and preoperative symptoms were comparable between N-HCC and A-HCC. As for surgical variables, N-HCC required less major liver resections (*P* = 0.005), which in turn resulted in less blood loss (*P* = 0.032). The surgical complication rate and in-hospital mortality rate were equivalent between the two groups. Nevertheless, the 6-month mortality or early mortality rate was significantly lower in N-HCC than in A-HCC (2.8% and 7.7%, respectively, *P* < 0.001) [29]. Considering pathological features, N-HCC tended to be smaller (*P* < 0.001), less vascular invaded (*P* < 0.001), and more well-differentiated (*P* < 0.001). They had less daughter nodules (*P* < 0.001), less cirrhosis (*P* = 0.002), and earlier T stage (*P* < 0.001) (Table 2). 

### 3.2. Survival Outcome of N-HCC After Hepatectomy

**Figure 1:**
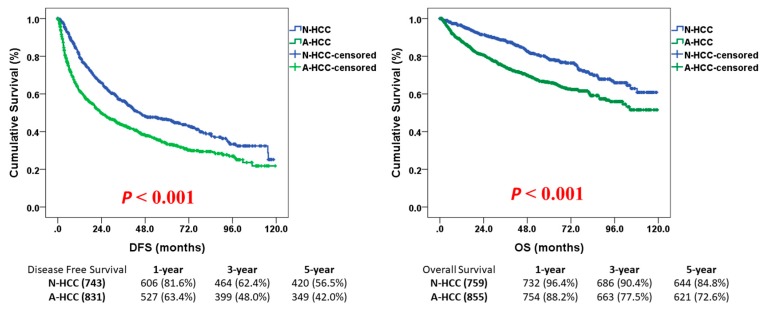
Kaplan–Meier disease-free survival (DFS) and overall survival (OS) curves for hepatocellular carcinoma with normal (N-HCC) or abnormal (A-HCC) AFP. A, Disease-free survival curves. The median DFS was 44.6 months (95% CI 34.2–54.9) for N-HCC and 23.6 months (95% CI 18.7–28.6) for A-HCC (*P* < 0.001). The one-, three-, and five-year DFS rates were 81.6%, 62.4%, and 56.5%, respectively, for N-HCC and 63.4%, 48.0%, and 42.0%, respectively, for A-HCC. B. Overall survival curves. The mean OS was 94.5 months (95% CI 91.0–97.9) for N-HCC and 81.7 months (95% CI 78.0–85.3) for A-HCC (*P* < 0.001). The one-, three-, and five-year OS rates were 96.4%, 90.4%, and 84.8%, respectively, for N-HCC and 88.2%, 77.5%, and 72.6%, respectively, for A-HCC. DFS, disease-free survival; OS, overall survival; N-HCC, normal α-fetoprotein HCC; A-HCC, abnormalα-fetoprotein HCC.

As for survival analysis, N-HCC had a significantly better disease-free survival (DFS) than A-HCC after hepatectomy. The median DFS was 44.6 months (95% CI 34.2–54.9) for N-HCC and 23.6 months (95% CI 18.7–28.6) for A-HCC (*P* < 0.001). The Kaplan-Meier DFS curves were illustrated in Figure 1. As shown in the figure, the one-, three-, and five-year DFS rates were 81.6%, 62.4%, and 56.5%, respectively, for N-HCC and 63.4%, 48.0%, and 42.0%, respectively, for A-HCC. The early recurrence rate was also significantly lower in N-HCC than in A-HCC (49.6% vs. 60.8%, respectively, *p* < 0.001). After univariate analysis, symptomatic diseases (anemia, jaundice, palpable mass, or ascites), ICG-15 greater than 10%, major liver resection, intraoperative blood loss more than 800 mL, operative duration more than 270 min, major complications, tumor size larger than 5 cm, ruptured tumor, vascular invasion, daughter nodules, and cirrhosis were found to be poor prognostic factors for DFS (all *P* < 0.05). Cox regression multivariate analysis further demonstrated that ICG-15 greater than 10% (HR 1.520, 95% CI 1.204–1.919, *P* < 0.001), tumor size larger than 5 cm (HR 1.823, 95% CI 1.366–2.433, *P* < 0.001), vascular invasion (HR 1.460, 95% CI 1.116–1.910, *P* = 0.006), daughter nodules (HR 1.565, 95% CI 1.182–2.072, *P* = 0.002), and histologically-proven cirrhosis (HR 1.272, 95% CI 1.008–1.605, *P* = 0.043) were independent poor prognostic factors for DFS in N-HCC (Table 3).

As for overall survival (OS), N-HCC still enjoyed a significantly longer OS than A-HCC after the operation. The mean OS was 94.5 months (95% CI 91.0–97.9) for N-HCC and 81.7 months (95% CI 78.0–85.3) for A-HCC (*P* < 0.001). The Kaplan–Meier OS curves were illustrated in Figure 1. As shown in Figure 1, the 1-, 3-, and 5-year OS rates were 96.4%, 90.4%, and 84.8%, respectively, for N-HCC and 88.2%, 77.5%, and 72.6%, respectively, for A-HCC. Univariate analysis identified that male gender, cigarette smoking, major liver resection, intraoperative blood loss more than 800 mL, operative duration more than 270 min, major complications, tumor size larger than 5 cm, vascular invasion, daughter nodules, cirrhosis, and necrosis were poor prognostic indicators for OS (all *P* < 0.05). Cox regression multivariate analysis further demonstrated that tumor size larger than 5 cm (HR 1.839, 95% 1.375–2.461, *P* < 0.001), vascular invasion (HR 1.549, 95% CI 1.190–2.015, *P* = 0.001), daughter nodules (HR 1.786, 95% CI 1.359–2.348, *P* < 0.001), and histologically-proven cirrhosis (HR 1.438, 95% CI 1.145–1.805, *P* = 0.002) were independent poor prognostic factors for OS in N-HCC after surgery (Table 4).

### 3.3. Identification of Potential Biomarkers for Normal AFP Hepatocellular Carcinoma

**Figure 2:**
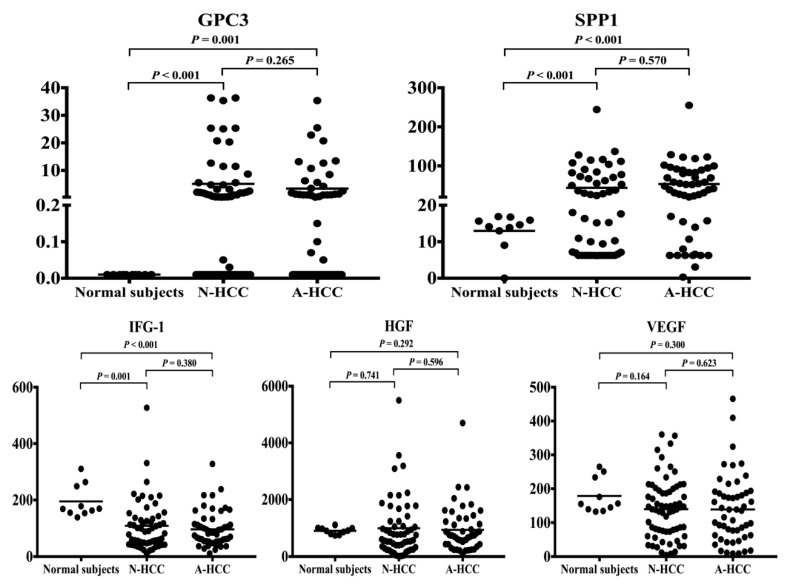
The scatter dot plots of GPC3, SPP1, IGF1, HGF, and VEGF. The serum levels of novel markers for N-HCC, A-HCC, and healthy subjects were determined by ELISA and represented as scatter dot plots. The arithmetic means of the tested parameters are indicated by a line. Student’s *t* test was employed for the statistical analysis and *P* < 0.05 was considered significant. GPC3, glypican 3; SPP1, secreted phosphoprotein 1; IGF1, insulin-like growth factor 1; HGF, hepatocyte growth factor; VEGF, vascular endothelial growth factor. N-HCC, normal α-fetoprotein HCC; A-HCC, abnormal α-fetoprotein HCC; ELISA, enzyme-linked immunosorbent assay.

Among the cohort of 147 HCC patients scheduled to receive curative hepatectomy, 74 patients (50.3%) had normal AFP levels. As shown in Table 5, N-HCC patients had significantly higher serum glypican 3 (GPC3) and secreted phosphoprotein 1 (SPP1, or osteopontin (OPN)) levels than healthy subjects (mean GPC3, 5.1 vs. <0.01; mean SPP1, 52.2 vs. 13.0). On the contrary, insulin-like growth factor 1 (IGF-1) was significantly lower in N-HCC patients (mean IGF-1, 104 vs. 195). The distributions of respective concentrations were shown in Figure 2. Since an effective tumor marker should be instinctively higher in cancer patients, we chose GPC3 and SPP1 for further analysis.

**Figure 3:**
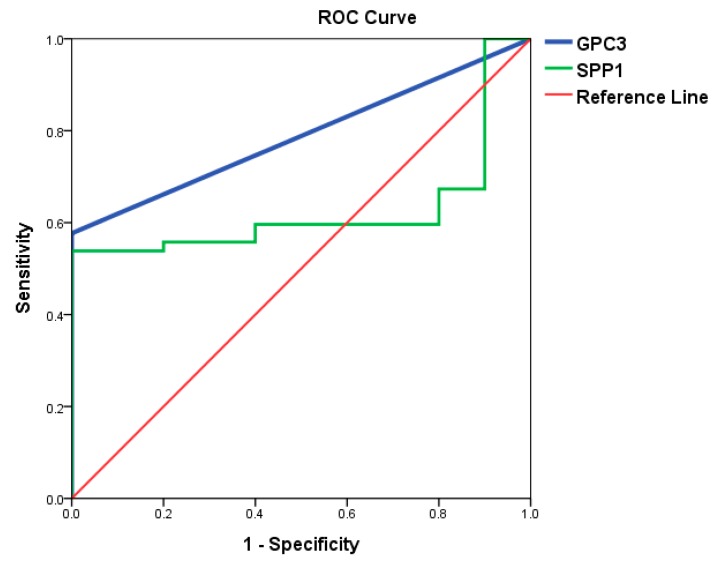
Performance of GPC3 and SPP1 ROC curves of GPC3 and SPP1 in differentiating N-HCC from healthy subjects. The area under the ROC curve of GPC3 and SPP1 for N-HCC was 0.788 (*P* = 0.004) and 0.625 (*P* = 0.213), respectively. GPC3, glypican 3; SPP1, secreted phosphoprotein 1; ROC, receiver operating characteristic.

As shown in Figure 3, the area under the ROC curve (AUC) of GPC3 and SPP1 for N-HCC was 0.788 (*P* = 0.004) and 0.625 (*P* = 0.213), respectively. GPC3 had a significantly better diagnostic capability for N-HCC than SPP1 in terms of AUC. When the cutoff value for GPC3 was set at 0.02 ng/mL, the sensitivity was 57.7% and the specificity of was 100% for N-HCC. On the other hand, when the cutoff value for SPP1 was set at 14.915 ng/mL, the sensitivity and specificity of OPN for N-HCC was only 59.6% and 60%, respectively. In other words, GPC3 may be a promising serum tumor marker for early detection and diagnosis of N-HCC.

**Figure 4:**
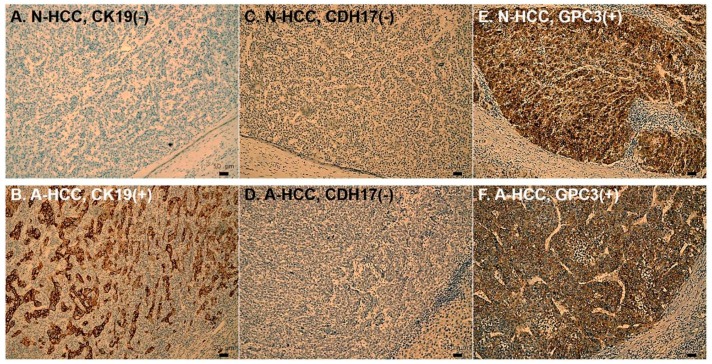
Immunohistochemical (IHC) microphotograph of primary HCC. The upper row (**A, C, E**) is IHC of N-HCC, while the lower row (**B, D, F**) is that of A-HCC. Most N-HCC and A-HCC (**E** and **F**) would express GPC3, while the expressions CDH17 were both low in N-HCC and A-HCC (**C** and **D**). CK19 was sparsely seen in N-HCC. About one-fourth of A-HCC tumors had CK19 expression (**B**); however, only 5% of N-HCC tumors expressed CK19 (**A**) (*P* = 0.003). (Magnifications, × 100). N-HCC, normal α-fetoprotein HCC; A-HCC, abnormalα-fetoprotein HCC; GPC3, glypican 3; CDH17, cadherin 17; CK19, cytokeratin 19.

**Figure 5:**
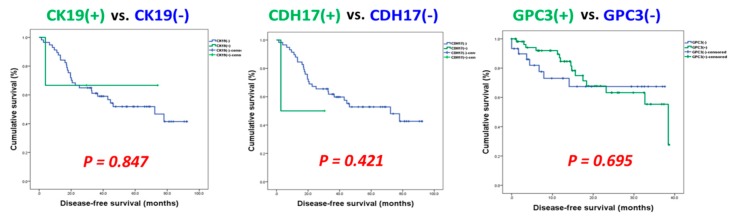
Kaplan–Meier disease-free survival (DFS) curves of N-HCC after hepatectomy Unlike HCC in general, CK19, CDH17, and GPC3 all were not significantly poor prognostic factors for DFS in N-HCC after hepatectomy (*P* all > 0.05). DFS, disease-free survival; N-HCC, normal α-fetoprotein HCC; GPC3, glypican 3; CDH17, cadherin 17; CK19, cytokeratin 19.

In addition to serum levels of GPC3, we also examined the expression profiles of GPC3 and other promising prognostic indicators in tumor samples of N-HCC. As shown in Figure 4 and Table S1, the immunohistochemical (IHC) study of N-HCC and A-HCC for expressions of cytokeratin 19 (CK19), cadherin 17 (CDH17), and GPC3 were analyzed and compared. In accordance with serum profiles, more than 65% of N-HCC tumors expressed GPC3, and around 75% of A-HCC tumors showed immunopositivity for GPC3. In the meantime, the expression profiles of CDH17 were comparable between N-HCC and A-HCC. On the contrary, CK19 was sparsely seen in N-HCC. Unlike A-HCC, in which one-fourth of tumors had CK19 expression, only 5% of N-HCC tumors expressed CK19 upon IHC examination (*P* = 0.003). Furthermore, the influence of these prognostic markers on oncological survival were also investigated in N-HCC. After statistical analysis, neither CK19, CDH17, nor GPC3 was found to be a significantly poor prognostic factor for disease-free survival (DFS) or overall survival (OS) (*P* all > 0.05) in N-HCC (Figure 5). 

## 4. Discussion

The current study demonstrated that normal AFP-HCC, or N-HCC, may be a distinct subclass of HCC based on evidence obtained from different aspects. First, the clinical analysis revealed that patients with N-HCC tended to be older males with less HBV infection, more non-viral etiology, and less cirrhosis. Since the vast majority of our non-viral N-HCC patients (87.8%) denied habitual alcohol consumption, carcinogenesis is, therefore, believed to be related to nonalcoholic fatty liver disease (NAFLD) or nonalcoholic steatohepatitis (NASH) [30,31]. This would implicate that NAFLD/NASH, in addition to viral hepatitis and cirrhosis, also plays an important role in the pathogenesis of N-HCC. According to a recent research, NASH may contribute to the development of HCC in the elderly without viral infection, and elderly HCC patients have significantly less liver cirrhosis [32]. Our study further indicates that these elderly non-viral HCC patients are more likely to be N-HCC. Secondly, the current study found that the N-HCC tended to be pathologically more well-differentiated and less advanced tumors. This would translate into a significantly better survival outcome in N-HCC than those with abnormal AFP levels after liver resection. Third, our translational study demonstrated that neither CK19, CDH17, nor GPC3 was found to be a significantly poor prognostic factor for either DFS or OS. This finding would contradict most of the previous publications that all CK19, CDH17, and GPC3 were poor prognostic indicators for HCC in general [33,34,35,36,37]. Lastly, we found in the current study that CK19, a notorious protein demonstrated to have worst outcome in HCC, was sparsely seen in N-HCC [33,34]. This and other results indicated that N-HCC may be a distinct subset of HCC that warrants further investigation. Unlike most of the previously published literature, which compared the outcome at a much higher AFP cut-off, our study employed the “normal” value as cut point and displayed the surgical as well as the long-term outcome. It is thus by far one of the largest series in the English literature to analyze the clinical-surgical-pathological variables and compare the survival outcome for HCC with normal AFP levels.

Many studies to date have been conducted to find new tumor markers for HCC. Nevertheless, most of them, instead of examining those HCC with lower or normal AFP levels, investigated HCC as a whole. The reported results, subsequently, may fail to discriminatively detect those HCC with normal AFP. It is thus of imperative significance to search for surrogate tumor markers for normal AFP-HCC. 

Glypican-3 (GPC3) is a membrane-bound heparan sulfate proteoglycan belonging to a family of six similar cell-surface proteins [38]. It is also a fetal protein and only trace amount can be detected in adult kidney. Recent studies have shown that GPC3 can be employed to differentiate HCC from non-malignant hepatocellular diseases in resected liver specimen by immunohistochemical staining [39,40,41]. Moreover, high serum GPC3 levels were reported to be diagnostic of HCC with high sensitivity and specificity [4,16,17]. Its’ role as a prognostic biomarker for HCC after liver resection was also demonstrated [36]. This and other evidence suggested that GPC3 could be a potentially promising tumor marker for the diagnosis of HCC. However, yet another recent study reported that the publications regarding serum GPC3 for HCC diagnostics could be flawed due to different patient selection, sample size, heterogeneous experimental technique, and serum quality control [42]. Furthermore, most studies examined HCC as a whole; few had tried to investigate the diagnostic performance of GPC3 for HCC with low or normal AFP [24]. Therefore, it is crucial to determine the actual value of GPC3 in the diagnosis of HCC, especially HCC with low AFP. The results obtained from the current study demonstrated, from both the serological and tissue levels, that GPC3 could be a promising serum marker for early detection of N-HCC. We believe, as a result, that AFP and GPC3 should be determined simultaneously in patients at risk to enhance the diagnostic accuracy of HCC.

In addition to being a diagnostic serum tumor marker, AFP was reported to have functional roles in HCC. Recent study suggested that AFP transcriptionally down-regulates miR-29a through action of c-MYC, which in turn activates DNA methyltransferase 3A gene expression and global epigenetic alterations, resulting in aggressive HCC behavior and poor prognosis [43]. In other words, not only a serum biomarker can become a promising diagnostic tumor marker, but also it may be functionally active in promoting tumor formation, invasion, and metastasis. Whether such tumor markers exist for N-HCC thus mandates further investigation.

Despite promising results, the present study still has several limitations. Firstly, incomplete or missing clinical data are inevitable when retrospectively reviewing medical records. Secondly, the treatment strategy for HCC may have evolved over the study period, which could potentially influence the study results. Studies with shorter recruiting duration may address this issue. Thirdly, the current study aimed to explore a serum marker for the diagnosis of N-HCC. For a more persuasive and significant result, a larger sample size comprising different populations of patients including those with HCC, chronic liver diseases, hepatitis, cirrhosis, and normal healthy subjects are mandatory. Last but not the least, a stringent external validation cohort is also warranted to confirm our findings. As a result, a large-scale prospective study comprising exploration set and external validation set should be conducted. 

## 5. Conclusions

In conclusion, the current study demonstrated that the clinicopathological characteristics of N-HCC were different from those of A-HCC in many aspects. N-HCC patients were significantly older with more comorbidities and less hepatitis virus infections. In addition, we found that GPC3 would be a promising tumor marker for diagnosing N-HCC. Our results implicate that the etiology and pathogenesis of N-HCC may be different from that of traditional HCC or A-HCC. This “N-HCC”, subsequently, should not be merely “normal AFP-HCC”; more specifically, we believe it should stand for “new category-HCC”. Further well-designed studies are warranted to validate our findings.

## Figures and Tables

**Table 1:** Clinical characteristics of normal alpha-fetoprotein hepatocellular carcinoma (N-HCC) ^a^ vs. abnormal alpha-fetoprotein hepatocellular carcinoma (A-HCC) ^b^ (*n* = 1616).

Variables ^c^	Total		N-HCC ^a^		A-HCC ^b^		*P*-Value ^d^
	No.	(%)	No.	(%)	No.	(%)	
Age > 65 year-old	577	(35.7%)	294	(38.6%)	283	(33.1%)	0.020
Male gender	1256	(77.7%)	619	(81.3%)	637	(74.5%)	0.001
Comorbidity	639	(39.9%)	339	(45.0%)	300	(35.4%)	<0.001
Diabetes	348	(21.7%)	198	(26.3%)	150	(17.7%)	<0.001
Hypertension	369	(34.7%)	185	(35.7%)	184	(33.7%)	0.490
ESRD ^e^	33	(2.1%)	23	(3.1%)	10	(1.2%)	0.008
HBV infection	878	(62.7%)	378	(56.2%)	500	(68.7%)	<0.001
HCV infection	471	(36.0%)	223	(35.7%)	248	(36.3%)	0.829
Non-B Non-C	207	(13.6%)	134	(18.6%)	73	(9.1%)	<0.001
Cigarette smoking	339	(21.0%)	169	(22.2%)	170	(19.9%)	0.252
Alcohol consumption	201	(12.4%)	100	(13.1%)	101	(11.8%)	0.419
Pre-OP symptoms ^f^	361	(22.3%)	164	(21.6%)	197	(23.0%)	0.473
ICG-15 ^g^ (%)	516	(34.0%)	238	(33.5%)	278	(34.5%)	0.664
Major procedure ^h^	444	(28.3%)	184	(24.9%)	260	(31.3%)	0.005
Blood loss >800 mL	188	(12.3%)	75	(10.4%)	113	(14.0%)	0.032
OP duration >270 min	672	(43.1%)	320	(43.5%)	352	(42.7%)	0.729
Major complication ^i^	153	(9.9%)	66	(9.0%)	87	(10.7%)	0.271
In-hospital mortality	26	(1.6%)	9	(1.2%)	17	(2.0%)	0.202
6-month mortality	87	(5.4%)	21	(2.8%)	66	(7.7%)	<0.001
	Mean	SEM ^j^	Mean	SEM ^i^	Mean	SEM ^i^	*P*-value
ICG-15 (%) ^g^	9.542	0.231	9.136	0.309	9.887	0.342	0.103
Hemoglobin (g/dL)	13.492	0.048	13.461	0.070	13.525	0.068	0.515
Albumin (g/dL)	4.112	0.012	4.136	0.018	4.093	0.016	0.083
Platelet (1000/uL)	179.268	1.826	179.199	2.569	178.779	2.617	0.909
ALT (U/L)	55.23	1.775	53.92	3.146	56.24	1.888	0.517
Bilirubin total (mg/dL)	0.764	0.013	0.751	0.022	0.775	0.014	0.341
Alkaline phosphatase (U/L)	93.59	2.378	94.76	4.609	92.64	1.894	0.661
α-fetoprotein (ng/mL) ^k^	14.40	222.60	4.60	4.60	227.20	1000.95	<0.001

^a^ Normal α-fetoprotein hepatocellular carcinoma; ^b^ Abnormal α-fetoprotein hepatocellular carcinoma ^c^ Only patients with available data were analyzed; ^d^ N-HCC vs. A-HCC; Pearson’s χ^2^ test was used to analyze the categorical variables, Student’s *t* test and Mann-Whitney U test were used to analyze continuous variables; ^e^ End-stage renal disease; ^f^ Include HCC presenting with anemia, jaundice, palpable mass, or ascites; ^g^ Indocyanine green retention test at 15 min; ^h^ Includes tri-segmentectomy, right/left lobectomy, and extended right/left lobectomy; ^i^ Includes grade III-IV surgical complications; ^j^ Standard error of mean; ^k^ Expressed as Median ± IQR.

**Table 2:** Pathologic characteristics of N-HCC ^a^ vs. A-HCC ^b^ (*n* = 1616).

Variables ^c^		Total		N-HCC ^a^		A-HCC ^b^		*P*-Value ^d^
		No.	(%)	No.	(%)	No.	(%)	
Tumor size (cm)	>5	505	(32.3%)	201	(27.3%)	304	(36.7%)	<0.001
Encapsulation	Yes	1282	(82.2%)	605	(82.7%)	677	(81.9%)	0.685
Capsular invasion	Yes	965	(62.0%)	408	(55.9%)	557	(67.4%)	<0.001
Rupture	Yes	120	(7.7%)	47	(6.4%)	73	(8.8%)	0.074
Vascular invasion	Yes	527	(33.8%)	178	(24.3%)	349	(42.3%)	<0.001
Daughter nodule	Yes	332	(21.3%)	109	(14.9%)	223	(27.0%)	<0.001
Cirrhosis	Yes	787	(50.4%)	338	(46.2%)	449	(54.2%)	0.002
Necrosis	Yes	754	(48.5%)	331	(45.4%)	423	(51.3%)	0.021
Edmondson-Steiner grading system	Grade 1/2	944	(61.8%)	527	(74.3%)	417	(50.9%)	<0.001
	Grade 3/4	584	(38.2%)	182	(25.7%)	402	(49.1%)	
T stage	T1	852	(58.1%)	456	(66.4%)	396	(50.8%)	<0.001
	T2	345	(23.5%)	152	(22.1%)	193	(24.7%)	
	T3a	133	(9.1%)	38	(5.5%)	95	(12.2%)	
	T3b	50	(3.4%)	6	(0.9%)	44	(5.6%)	
	T4	87	(5.9%)	35	(5.1%)	52	(6.7%)	
N stage	N1	12	(0.8%)	4	(0.6%)	8	(1.0%)	0.343

^a^ Normal α-fetoprotein hepatocellular carcinoma ^b^ Abnormal α-fetoprotein hepatocellular carcinoma ^c^ Only patients with available data were analyzed ^d^ N-HCC vs. A-HCC; Pearson’s χ^2^ test was used to analyze the categorical variables.

**Table 3:** Univariate and cox regression multivariate analyses of factors associated with disease-free survival (DFS) ^a^ in N-HCC ^b^ after hepatectomy.

Variables ^c^	Univariate		Multivariate	
	Median DFS ^a^ ± SE (months)	*P*-Value	Hazard Ratio (95% CI)	*P*-Value
Age (>65 vs. ≤65 (year-old))	39.4 ± 4.6 vs. 49.2 ± 8.6	0.131		
Gender (male vs. female)	41.9 ± 5.8 vs. 70.6 ± 15.7	0.115		
Diabetes mellitus (yes vs. no)	45.6 ± 12.0 vs. 44.6 ± 6.3	0.428		
Hypertension (yes vs. no)	41.7 ± 11.5 vs. 41.6 ± 5.0	0.523		
ESRD ^d^ (yes vs. no)	76.0 ± 29.0 vs. 44.6 ± 5.4	0.873		
HBV surface antigen (positive vs. negative)	47.9 ± 7.4 vs. 41.0 ± 7.1	0.310		
Hepatitis C virus (positive vs. negative)	32.9 ± 5.2 vs. 46.2 ± 8.0	0.182		
Cigarette smoking (yes vs. no)	37.6 ± 5.6 vs. 47.0 ± 7.0	0.197		
Alcohol consumption (yes vs. no)	41.9 ± 11.8 vs. 45.6 ± 6.3	0.526		
Pre-OP symptoms ^e^ (yes vs. no)	31.8 ± 5.4 vs. 60.0 ± 7.5	0.004	1.278 (0.986–1.657)	0.064
ICG-15 ^f^ (>10 vs. ≤10 (%))	32.7 ± 4.8 vs. 57.2 ± 7.8	0.002	1.520 (1.204–1.919)	<0.001
Procedure type (Major ^g^ vs. Minor)	28.2 ± 7.0 vs. 61.4 ± 8.0	<0.001	1.017 (0.751–1.377)	0.913
Blood loss (>800 vs. ≤800 (mL))	30.7 ± 11.0 vs. 47.0 ± 6.3	0.034	1.095 (0.750–1.597)	0.639
OP duration (>270 vs. ≤270 (mins))	36.7 ± 4.2 vs. 70.6 ± 9.1	<0.001	1.238 (0.964–1.591)	0.094
Complication (Major ^h^ vs. Minor/none)	27.7 ± 10.7 vs. 46.2± 6.2	0.044	1.031 (0.699–1.520)	0.879
Albumin (≤3.5 vs. >3.5 (g/dL))	40.1 ± 5.6 vs. 47.0 ± 6.3	0.175		
Tumor size (>5 vs. ≤5 (cm))	26.9 ± 4.6 vs. 67.9 ± 8.7	<0.001	1.823 (1.366–2.433)	<0.001
Capsule (yes vs. no)	47.0 ± 6.2 vs. 32.9 ± 5.9	0.147		
Capsular invasion (yes vs. no)	41.9 ± 7.7 vs. 49.2 ± 9.2	0.264		
Rupture (yea vs. no)	32.0 ± 11.9 vs. 47.9 ± 6.7	0.010	1.098 (0.709–1.701)	0.676
Vascular invasion (yes vs. no)	22.0 ± 4.2 vs. 63.5 ± 8.9	<0.001	1.460 (1.116–1.910)	0.006
Daughter nodule (yes vs. no)	22.2 ± 2.6 vs. 61.4 ± 7.5	<0.001	1.565 (1.182–2.072)	0.002
Cirrhosis (yes vs. no)	40.1 ± 3.8 vs. 62.3 ± 9.6	0.040	1.272 (1.008–1.605)	0.043
Necrosis (yes vs. no)	40.6 ± 3.8 vs. 63.5 ± 8.8	0.061		
Edmondson–Steiner grading system(grade 3/4 vs. grade 1/2)	37.4 ± 7.6 vs. 47.0 ± 6.2	0.101		

^a^ Disease-free survival ^b^ Normal α-fetoprotein hepatocellular carcinoma ^c^ Only patients with available data were analyzed ^d^ End-stage renal disease ^e^ Include HCC presenting with anemia, jaundice, palpable mass, or ascites ^f^ Indocyanine green retention test at 15 min ^g^ Includes tri-segmentectomy, right/left lobectomy, and extended right/left lobectomy ^h^ Includes grade III-IV surgical complications.

**Table 4:** Univariate and cox regression multivariate analyses of factors associated with OS ^a^ in N-HCC ^b^ after hepatectomy.

Variables ^c^	Univariate		Multivariate	
	Mean OS ^a^ ± SE (Months)	*P*-Value	Hazard Ratio (95% CI)	*P*-Value
Age (>65 vs. ≤65 (year-old))	90.7 ± 3.2 vs. 96.6 ± 2.1	0.084		
Gender (male vs. female)	92.7 ± 2.0 vs. 102.8 ± 3.7	0.037	1.153 (0.838–1.586)	0.382
Diabetes mellitus (yes vs. no)	88.0 ± 3.8 vs. 96.4 ± 2.0	0.223		
Hypertension (yes vs. no)	98.8 ± 3.8 vs. 94.1 ± 2.9	0.841		
ESRD ^d^ (yes vs. no)	70.8 ± 4.2 vs. 95.1 ± 1.8	0.984		
HBV surface antigen (positive vs. negative)	94.1 ± 2.4 vs. 98.2 ± 2.9	0.501		
Hepatitis C virus (positive vs. negative)	96.9 ± 3.4 vs. 91.7 ± 2.5	0.189		
Cigarette smoking (yes vs. no)	88.8 ± 4.3 vs. 96.2 ± 1.9	0.032	1.075 (0.819–1.411)	0.604
Alcohol consumption (yes vs. no)	95.2 ± 4.6 vs. 94.6 ± 1.9	0.986		
Pre-OP symptoms ^e^ (yes vs. no)	91.8 ± 3.8 vs. 95.4 ± 2.0	0.129		
ICG-15 ^f^ (>10 vs. ≤10 (%))	89.6 ± 3.5 vs. 95.4 ± 2.1	0.120		
Procedure type (Major ^g^ vs. Minor)	80.1 ± 3.6 vs. 98.7 ± 1.9	<0.001	1.023 (0.760–1.376)	0.881
Blood loss (>800 vs. ≤800 (mL))	71.4 ± 6.0 vs. 97.0 ± 1.8	<0.001	1.131 (0.779–1.643)	0.516
Complication (Major ^h^ vs. Minor/none)	66.3 ± 5.6 vs. 97.2 ± 1.8	<0.001	1.118 (0.762–1.639)	0.569
Albumin (≤3.5 vs. >3.5 (g/dL))	79.6 ± 5.2 vs. 95.3 ± 1.9	0.064		
Tumor size (>5 vs. ≤5 (cm))	82.4 ± 3.7 vs. 99.1 ± 1.9	<0.001	1.839 (1.375–2.461)	<0.001
Capsule (yes vs. no)	94.8 ± 1.9 vs. 93.9 ± 4.4	0.709		
Capsular invasion (yes vs. no)	92.9 ± 2.4 vs. 96.2 ± 2.7	0.287		
Rupture (yea vs. no)	85.7 ± 5.9 vs. 94.9 ± 1.8	0.585		
Vascular invasion (yes vs. no)	78.8 ± 4.2 vs. 99.5 ± 1.9	<0.001	1.549 (1.190–2.015)	0.001
Daughter nodule (yes vs. no)	75.9 ± 4.7 vs. 98.0 ± 1.9	<0.001	1.786 (1.359–2.348)	<0.001
Cirrhosis (yes vs. no)	90.3 ± 2.7 vs. 98.1 ± 2.3	0.046	1.438 (1.145–1.805)	0.002
Necrosis (yes vs. no)	90.9 ± 2.6 vs. 97.9 ± 2.4	0.049	1.132 (0.889–1.442)	0.315
Edmondson–Steiner grading system (grade 3/4 vs. grade 1/2)	90.1 ± 3.7 vs. 96.0 ± 2.0	0.068		

^a^ Overall survival ^b^ Normal α-fetoprotein hepatocellular carcinoma ^c^ Only patients with available data were analyzed ^d^ End-stage renal disease ^e^ Include HCC presenting with anemia, jaundice, palpable mass, or ascites ^f^ Indocyanine green retention test at 15 min ^g^ Includes tri-segmentectomy, right/left lobectomy, and extended right/left lobectomy ^h^ Includes grade III-IV surgical complications.

**Table 5:** Serum concentrations of various markers in patients with HCC (*n* = 147).

Variables	N-HCC ^a^ vs. A-HCC ^b^		N-HCC ^a^ vs. Healthy Subject		A-HCC ^b^ vs. Healthy Subject	
	Mean ± SEM	*P*-Value ^h^	Mean ± SEM	*P*-Value ^h^	Mean ± SEM	*P*-Value ^h^
GPC3 ^c^ (ng/mL)	5.1± 1.2 vs. 3.4 ± 0.9	0.265	5.1± 1.2 vs. < 0.01	<0.001	3.4± 0.9 vs. < 0.01	0.001
SPP1 ^d^ (ng/mL)	52.2 ± 8.3 vs. 58.2 ± 6.5	0.570	52.2 ± 8.3 vs. 13.0 ± 1.6	<0.001	58.2 ± 6.5 vs. 13.0 ± 1.6	<0.001
IGF-1 ^e^ (ng/mL)	104 ± 11 vs. 92 ± 8	0.380	104 ± 11 vs. 195 ± 18	0.001	91.7± 7.8 vs. 195 ± 18	<0.001
HGF ^f^ (pg/mL)	952 ± 123 vs. 1043 ± 120	0.596	952 ± 123 vs. 909 ± 38	0.741	1043 ± 120 vs. 909 ± 38	0.292
VEGF ^g^ (pg/mL)	137 ± 12 vs. 145 ± 13	0.623	137 ± 12 vs. 179 ± 16	0.164	145 ± 12 vs. 179 ± 16	0.300

^a^ Normal α-fetoprotein hepatocellular carcinoma ^b^ Abnormal α-fetoprotein hepatocellular carcinoma ^c^ Glypican-3 ^d^ secreted phosphoprotein 1 (SPP1), also known as osteopontin (OPN) ^e^ Insulin-like growth factor 1 ^f^ Hepatocyte growth factor ^g^ Vascular endothelial growth factor ^h^ Student’s *t* test.

## Supplementary Materials

The following are available online at https://www.mdpi.com/2077-0383/8/10/1736/s1, Table S1: IHC characteristics of N-HCC vs. A-HCC.
