# Supplementary material for: Normal Alpha-Fetoprotein Hepatocellular Carcinoma: Are They Really Normal?
Source: J Clin Med. 2019 Oct 19;8(10):1736. doi: 10.3390/jcm8101736 (PMC6832124; doi:10.3390/jcm8101736)
Supplement: Supplementary file 1 [file jcm-08-01736-s001.pdf]

Table S1 – IHC[a] characteristics of N-HCC[b] *vs*. A-HCC[c] (n=114)

Variables		Total		N-HCC[b]		A-HCC[c]		*P*-value [g]
		No.	(%)	No.	(%)	No.	(%)	
**GPC3[d]**	Positive	80	(70.2%)	44	(65.7%)	36	(76.6%)	**0.173**
	Negative	34	(29.8%)	23	(34.3%)	11	(23.4%)	
**CDH17[e]**	Positive	6	(5.3%)	3	(4.9%)	3	(5.7%)	**0.859**
	Negative	108	(94.7%)	58	(95.1%)	50	(94.3%)	
**CK19[f]**	Positive	16	(14.0%)	3	(5.0%)	13	(24.1%)	**0.003**
	Negative	98	(86.0%)	57	(95.0%)	41	(75.9%)	

[a] Immunohistochemical study

[b] Normal α-fetoprotein hepatocellular carcinoma

[c] Abnormal α-fetoprotein hepatocellular carcinoma

[d] Glypican-3

[e] Cadherin-17

[f] Cytokeratin-19

[g] N-HCC *vs.* A-HCC; Pearson's $\chi 2$ test was used to analyze the categorical variables
